# Karyotype differentiation in tellin shells (Bivalvia: Tellinidae)

**DOI:** 10.1186/s12863-017-0535-1

**Published:** 2017-07-14

**Authors:** Daniel García-Souto, Gonzalo Ríos, Juan J. Pasantes

**Affiliations:** Departamento de Bioquímica, Xenética e Inmunoloxía, Universidade de Vigo, E-36310 Vigo, Spain

**Keywords:** Tellin shells, Chromosome, Fluorescent in situ hybridization, Histone genes, Ribosomal RNA genes

## Abstract

**Background:**

Although Tellinidae is one of the largest and most diverse families of bivalves, its taxonomy is utterly chaotic. This is mainly due to the morphological diversity and homoplasy displayed by their shells and to the scarcity of the molecular phylogenetic studies performed on them. A molecular cytogenetic analysis of four tellin shell species, *Bosemprella incarnata*, *Macomangulus tenuis*, *Moerella donacina* and *Serratina serrata*, was performed. To molecularly characterize the analyzed specimens, the sequence of a fragment of the mitochondrial cytochrome c oxidase subunit I (COI) was also studied.

**Results:**

The karyotypes of the four species were composed of different amounts of bi-armed and telocentric chromosomes. The chromosomal mapping of 45S and 5S rDNA and H3 histone gene clusters by fluorescent in situ hybridization also revealed conspicuous differences on the distribution of these DNA sequences on their karyotypes. Vertebrate type telomeric sequences were located solely on both ends of each chromosome in all four tellin shells.

**Conclusion:**

We present clear evidence of the valuable information provided by FISH signals in both analyzing chromosome evolution in Tellinidae and as a further tool in identifying tellin shell specimens for molecular phylogenies.

## Background

Tellin shells are fast-burrowing bivalves inhabiting marine and estuarine soft bottom ecosystems; they are distributed worldwide but are particularly abundant at tropical latitudes [1]. With over 550 living species organized in nine subfamilies and 107 genera, Tellinidae (Blainville, 1814) is one of the largest and most diverse families of bivalves [2]. In spite of that, the taxonomy of tellin shells is utterly chaotic, mainly as a consequence of the morphological diversity and homoplasy displayed by their shells [1, 2]. The situation is further aggravated by the scarcity of molecular phylogenetic studies on this family [2]. The few molecular analyses including *Limecola* and *Serratina* sequences [3–5] have also challenged traditional systematics and demonstrated improper placement and/or misidentification of some of the analyzed specimens. Therefore, to overcome these difficulties and clarify the classification of tellin shells further identification criteria are required in order to integrate traditional cladistic and molecular phylogenetic studies.

Although chromosome analyses have helped in both characterizing and solving identification problems in some groups of bivalves [6, 7], cytogenetic data in tellin shells is rather scarce and mostly limited to the description of diploid chromosome numbers (2*n* = 38) and karyotypes in three species, *Macomangulus tenuis* [8], *Limecola balthica* [8, 9] and *Macoma nasuta* [10], and to the location of the nucleolar organizing regions (NORs) by silver staining in *L. balthica* [11] and by fluorescent in situ hybridization (FISH) in *M. nasuta* [10]. Additionally, chromosome analysis has also been applied to study neoplastic cells in *L. balthica* [11–14].

To verify if a cytogenetic approach can help in solving some of the identification problems in tellin shells, in this work we characterized the chromosomes of four Tellinidae taxa by means of chromomycin A3 (CMA), 4′,6-diamidino-2-phenylindole (DAPI) and propidium iodide (PI) fluorescence staining and FISH mapping telomeric sequences and 45S rDNA, 5S rDNAs and H3 histone gene clusters. A fragment of the mitochondrial cytochrome c oxidase subunit I (COI) gene was also amplified and sequenced in the same tellin shell specimens.

## Methods

### Tellin shell specimens

**Fig. 1:**
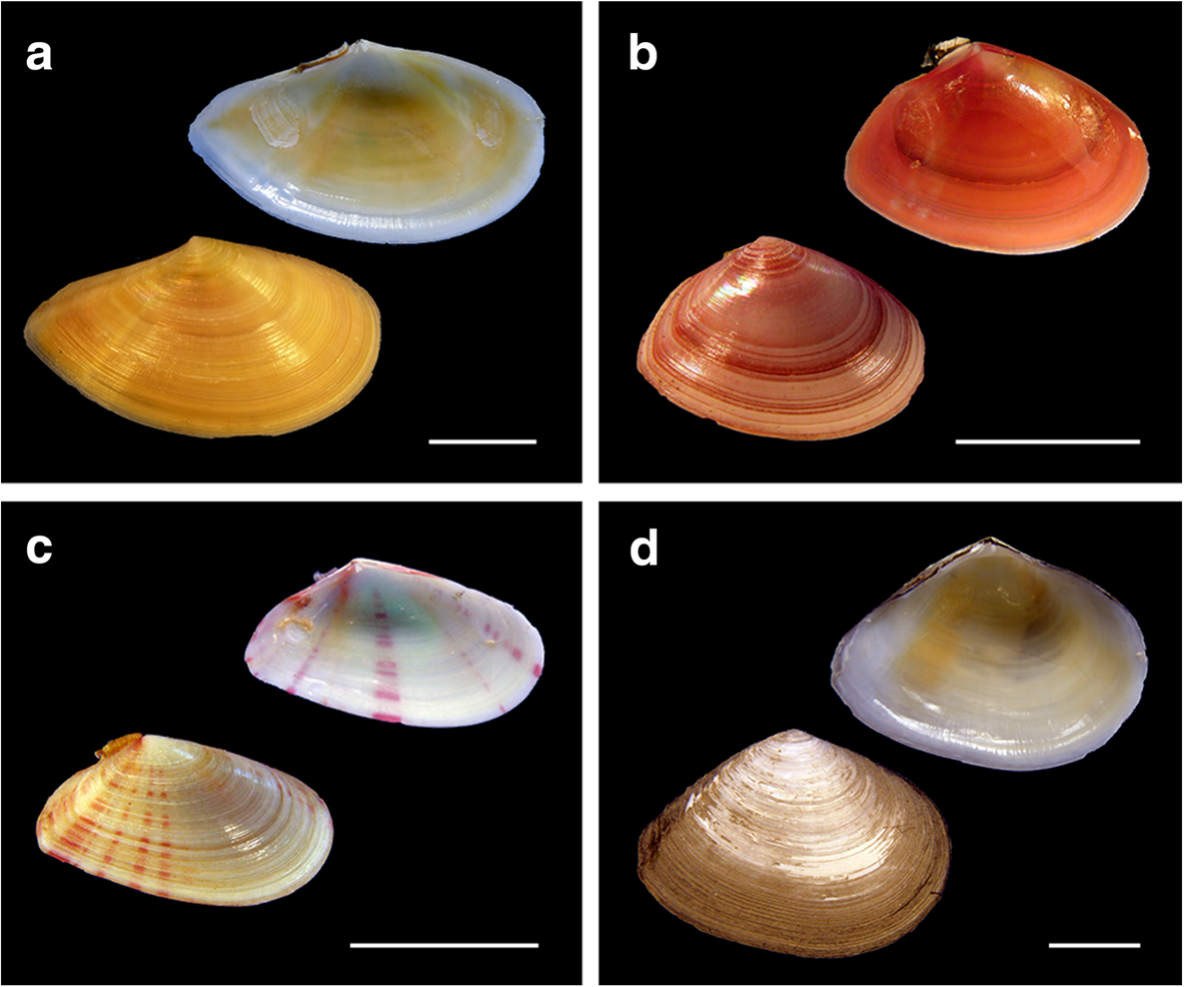
Shells of studied specimens of the family Tellinidae. Representative shells of (**a**) *Bosemprella incarnata*, (**b**) *Macomangulus tenuis*, (**c**) *Moerella donacina* and (**d**) *Serratina serrata*. Scale bars, 1 cm

Tellin shell specimens were collected in Ría de Pontevedra (NW Spain). The specimens were identified as *Moerella donacina* (Linnaeus, 1758), *Serratina serrata* (Brocchi, 1814), *Macomangulus tenuis* (da Costa, 1778) and *Bosemprella incarnata* (Linnaeus, 1758) according to shell morphology criteria. Representative specimens of the four taxa studied appear in Fig. 1. The nomenclature used for the taxa follows the World Register of Marine Species database (http://www.marinespecies.org/). To promote somatic growth, tellin shells were maintained in adequate laboratory conditions for 1 week [15].

### Chromosome preparation and fluorochrome staining

Following an overnight colchicine (0.005%) treatment, tellin shells were euthanized and gill and gonadic tissues dissected and immersed in 50% (20 min) and 25% (20 min) sea water prior to fixation with ethanol/acetic acid following previously described methods for cytogenetic studies in bivalves [16]. Chromosome spreads were obtained by disaggregating small pieces of tissue in 60% acetic acid and dropping the resulting cellular suspension onto preheated slides [17, 18].

After verifying the quality of the preparations by phase contrast microscopy, sequential fluorochrome staining was then performed [19–21]. Briefly, chromosomes were stained with CMA (0.25 mg/mL, 2 h) and DAPI (0.14 μg/mL, 8 min), mounted with antifade (Vectashield, Vector) and examined by fluorescent microscopy. For every single metaphase plate, images for each fluorochrome were acquired, pseudocolored and merged. After washing and staining with DAPI and PI (0.07 μg/mL, 8 min), the same metaphase plates were recorded again.

### DNA isolation, PCR amplification and probe labeling

Genomic DNA was extracted with the EZNA Mollusc DNA Kit (Omega) following manufacturer’s instructions. A BioDrop μLITE (Biodrop) was employed to assess the purity and the concentration of the genomic DNA samples.

FISH probes were obtained by amplifying a 28S rDNA fragment with primers *LR10R* and *LR12* [22], the entire 5S rDNA repeat with primers described by Pérez-García et al. [19] and the H3 histone gene with those reported by Giribet and Distel [23]. To molecularly characterize the studied tellin shells, a fragment of the mitochondrial COI gene was amplified in all karyotyped specimens using the primers developed by Nantón et al. [24] for Donacidae.

PCRs were carried out in a GeneAmp PCR system 9700 (Applied Biosystems) in 20 μL volumes containing 50 ng DNA, 1× reaction buffer, 2.5 mM MgCl_2_, 0.5 mM each dNTP (Thermo Fisher Scientific), 1 μM each primer and 1 U BIOTAQ DNA polymerase (Bioline). The amplification of the 28S rDNA included 30 cycles of 20 s denaturation at 95 °C, 20 s annealing at 48 °C and 30 s extension at 72 °C. For 5S rDNA, 40 cycles (95 °C, 44 °C and 72 °C, 20 s each) of amplification were employed. H3 histone gene amplification used 30 cycles (95 °C, 48 °C and 72 °C for 15 s each). For COI gene 35 amplification cycles (95 °C, 48 °C and 72 °C, 30 s each) were employed. All PCR reactions included an initial denaturation at 95 °C (10 min) and a final extension at 72 °C (5 min).

28S rDNA fragments were labeled either with biotin-16-dUTP (Roche) or digoxigenin-11-dUTP (Roche) by nick translation. Labeling of 5S rDNA and H3 histone gene probes was directly performed by supplementing the PCR mixture with either biotin-16-dUTP (20 μM) or digoxigenin-11-dUTP (5 μM).

### DNA sequencing

COI PCR products were purified (FavorPrep™ GEL/PCR Purification Kit, Favorgen) and those corresponding to two specimens per taxa were sequenced in both directions using a BigDye terminator kit v3.1 (Applied Biosystems) in an ABI PRISM 3730 Genetic Analyzer (Applied Biosystems) by the genomic service (CACTI) of the University of Vigo. DNA sequences were edited with BioEdit v. 7.1.11 [25], aligned with MUSCLE in MEGAv7 [26] and trimmed to 545 bp after excluding primers.

### Fluorescent in situ hybridization (FISH)

Single, double and sequential FISH were performed following previously described protocols [21, 27, 28]. Chromosome spreads were digested with RNase and pepsin before denaturating them in 70% formamide (70 °C, 2 min). Hybridization was performed overnight at 37 °C. Biotin detection was carried out with fluorescein isothiocyanate (FITC) conjugated avidin and biotinylated anti-avidin (Vector), whilst probes labeled with digoxigenin were detected with mouse anti-digoxigenin and anti-mouse tetramethylrhodamine (TRITC) antibodies. Chromosome preparations were counterstained with DAPI. After employing a commercial (C_3_TA_2_)_3_ peptide nucleic acid (PNA) probe (Applied Biosystems) for FISH mapping vertebrate telomeric repeats, chromosome preparations were counterstained with DAPI and PI. As indicated above, separated images for each fluorochrome were recorded, pseudo-colored and merged. For karyotyping and mapping purposes, at least 10 specimens per taxa (5 female, 5 male) and 20 metaphase plates per specimen were surveyed.

## Results

**Fig. 2:**
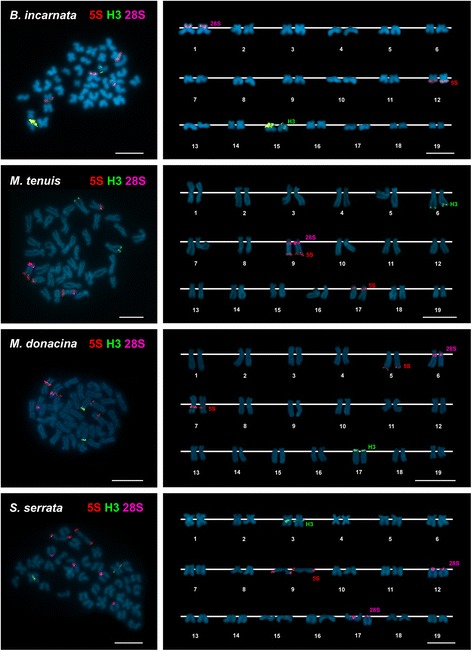
Mapping of rDNA and histone gene clusters to the chromosomes of four species of tellin shells counterstained with DAPI. FISH mapping of 5S rDNA (5S, *red*), 28S rDNA (28S, *magenta*) and H3 histone gene (H3, *green*) probes to mitotic metaphase plates, and corresponding karyotypes, of *Bosemprella incarnata*, *Macomangulus tenuis*, *Moerella donacina* and *Serratina serrata*. Scale bars, 5 μm

All specimens of *Bosemprella incarnata*, *Macomangulus tenuis*, *Moerella donacina* and *Serratina serrata* are 2n = 38. As shown in Fig. 2, these tellin shells present diverse karyotype compositions. *Bosemprella incarnata* exhibits three metacentric, nine submetacentric, two subtelocentric and five telocentric chromosome pairs. *Macomangulus tenuis* karyotype is composed of one metacentric, eight submetacentric and ten subtelocentric pairs. In *Moerella donacina* seven metacentric, five submetacentric, six subtelocentric and one telocentric chromosome pairs appear. Lastly, *Serratina serrata* showed a karyotype composed of four metacentric, six submetacentric, three subtelocentric and six telocentric chromosome pairs.

The combined use of AT-specific (DAPI), GC-specific (CMA) and unspecific fluorochromes revealed DAPI-dull/CMA-bright, GC-rich regions close to the centromeres in a single chromosome pair in *Bosemprella incarnata*, *Macomangulus tenuis* and *Moerella donacina*, and in two chromosome pairs in *Serratina serrata*. FISH experiments employing a 28S rDNA probe (Fig. 2) demonstrated that these GC-rich regions are coincident with the location of the 45S rDNA clusters.

A single H3 histone gene cluster was present in all four tellin shell species analyzed (Fig. 2). This cluster was subcentromeric to the short arms of both telocentric chromosome pair 15 in *Bosemprella incarnata* and submetacentric chromosome pair 3 in *Serratina serrata* and to the short arms of telocentric pair 17 in *Moerella donacina*. In contrast, H3 histone genes were subterminal to the long arms of subtelocentric pair 6 in *Macomangulus tenuis*.

Single 5S rDNA clusters mapped (Fig. 2) to subterminal positions on the long arms of metacentric pair 12 in *Bosemprella incarnata* and telocentric pair 9 in *Serratina serrata*. The remaining two tellin shells presented two 5S rDNA clusters. Subterminal to the long arms of telocentric pair 9 and subcentromeric to the long arms of subtelocentric pair 17 in *Macomangulus tenuis*; subterminal to the long arms of subtelocentric pair 5 and subcentromeric to the long arms of metacentric pair 7 in *Moerella donacina*.

Double FISH experiments using 28S and 5S rDNA probes labeled differently demonstrated that subtelocentric chromosome pair 9 of *Macomangulus tenuis* bears both subcentromeric 45S rDNA and subtelomeric 5S rDNA clusters in its long arms.

FISH analysis employing a telomeric (C_3_TA_2_)_3_ PNA probe revealed terminal signals at both ends of every chromosome in the four tellin shells.

**Fig. 3:**
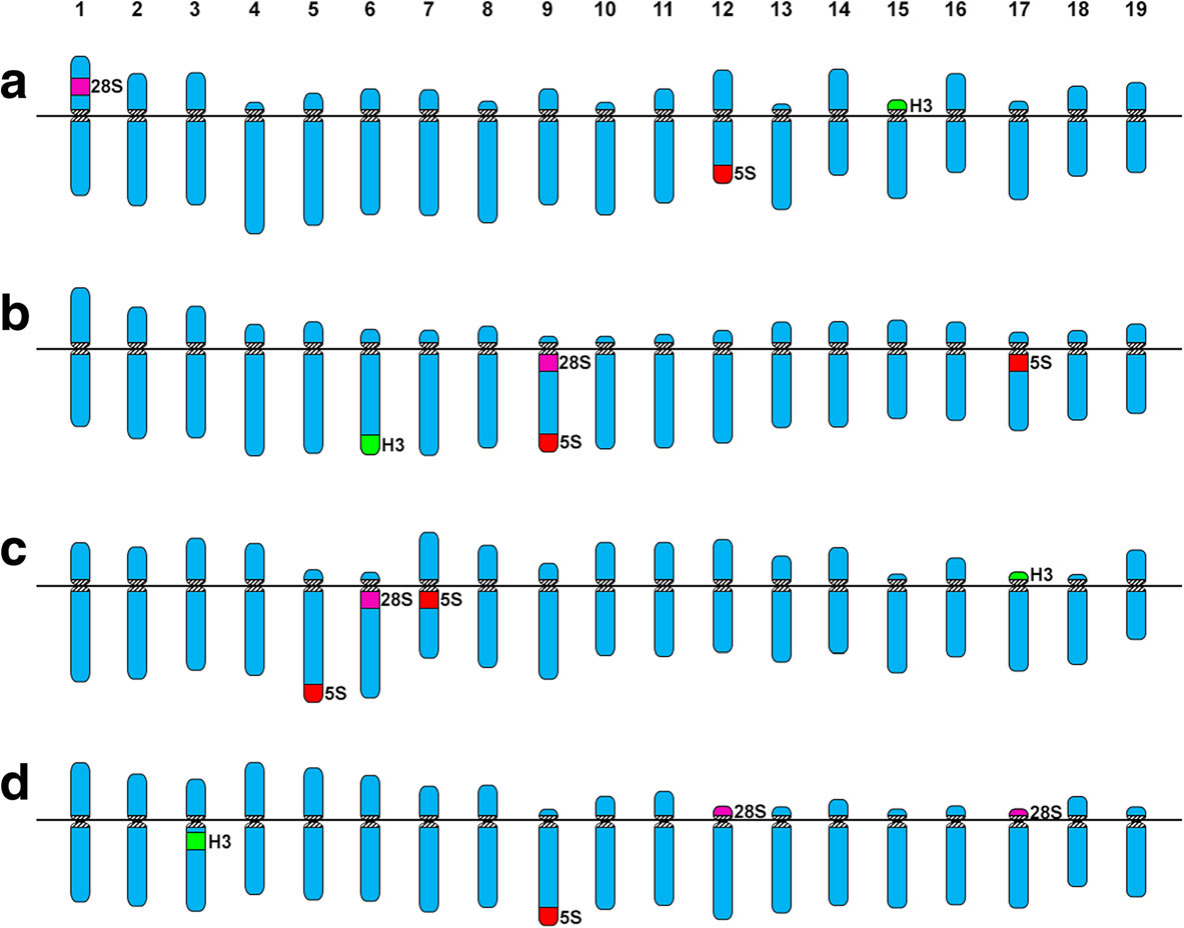
Ideogrammatic karyotypes of tellin shells. Schematic representation of the karyotypes of (**a**) *Bosemprella incarnata*, (**b**) *Macomangulus tenuis*, (**c**) *Moerella donacina* and (**d**) *Serratina serrata*. The schematic representation of the chromosomal mapping results shows H3 histone genes in green, 5S rDNA in red and 45S rDNA (NORs) in magenta

A schematic representation of the cytogenetic results obtained is showed in Fig. 3 and a summary of the published cytogenetic data for Tellinidae species is presented in Table 1.

**Table 1:** Karyotype composition and chromosomal mapping of rDNAs and histone genes in Tellinidae

		Karyotype composition							
	*n*	(m)	(sm)	(st)	(t)	45S rDNA	5S rDNA	*h3*	References
*Bosemprella incarnata*	19	3	9	2	5	1p ic (m)	12q ter (m)	15p cen (t)	This work
*Limecola balthica*	19	13	1	5					[8]
	19	11	2	6					[9]
	19	11	2	6		1q cen (m)			[11]
						4q ter (m)			
						?q ter (st)			
*Macoma nasuta*	19	8	6	5		15q ter (st)			[10]
*Macomangulus tenuis*	19	2	5	12					[8]
	19	1	8	10		9q cen (st)	9q ter (st)	6q ter (st)	This work
							17p cen (st)		
*Moerella donacina*	19	7	5	6	1	6q cen (st)	5q ter (st)	17p cen (t)	This work
							7q ic (m)		
*Serratina serrata*	19	4	6	3	6	12p cen (t)	9q ter (t)	3q cen (m)	This work
						17p cen (t)			

*Abbreviations*: (*m*): metacentric, (*sm*) submetacentric, (*st*) subtelocentric, (*t*) telocentric,? not described, *p*: short arm, *q* long arm, *cen* subcentromeric, *ic* intercalary, *ter* subterminal

A fragment of the mitochondrial COI gene was successfully amplified in the four Tellinidae taxa and the corresponding sequences stored in GenBank under accession numbers KY951457 and KY951458 for *Bosemprella incarnata*, KY951461 and KY951462 for *Macomangulus tenuis*, KY951455 and KY951456 for *Moerella donacina* and KY951459 and KY951460 for *Serratina serrata*. All partial COI gene sequences were independently compared using BLAST with those stored in NCBI GenBank and BOLD databases. Out of these, high similarity levels (>99%) were only found among our *Macomangulus tenuis* sequences (Genbank accession numbers KY951461 and KY951462) and a single sequence (Genbank accession number KR084511) from a North Sea specimen stored under the same specific assignation. The same comparison approaches using all the other sequences obtained in this work always displayed levels of similarity (<93%) well below those usually corresponding to the same taxa in bivalves.

## Discussion

The diploid chromosome numbers, 2n = 38, found for the four tellin shell species studied here coincide with those previously reported for *Macomangulus tenuis* [8] and the other two Tellinidae species previously described, *Limecola balthica* [8, 9] and *Macoma nasuta* [10]. This is also the usual diploid number in most Heterodonta bivalve species [21, 28–36].

In regards to karyotype composition, the main difference with previous data for Tellinidae (Table 1) is the occurrence of telocentric chromosome pairs in three of the four tellin shells studied here, a relatively unusual condition in Heterodonta [34]. This is not the case for *Macomagulus tenuis* whose karyotype, in concordance with previous results [8], is exclusively composed of bi-armed chromosomes; the minor differences between the karyotype composition reported here and the previously published one (Table 1) can be attributed to methodological variation.

As in most other bivalves studied to date [6, 7, 19–21, 27, 28, 31–36], the application of diverse combinations of base-specific fluorochromes to the staining of tellin shell chromosomes demonstrated that GC-rich regions are scarce, two in *Serratina serrata* and a single one in the other three species. As is also the rule for most bivalve species, further FISH using 28S rDNA probes confirmed that these GC-rich regions are coincident with the chromosomal regions bearing 45S rDNA clusters, the NORs.

Tandemly repeated DNA sequences are helpful markers for identifying chromosomes. This is also the case in bivalves in which FISH mapping made possible establishing more reliable karyotypes and identifying some of the chromosomal changes accompanying bivalve evolution [6]. As shown in Table 1, this kind of approach has barely been applied to tellin shells. Only 45S rDNAs have been previously located, either by silver staining (*Limecola balthica*) [11] or FISH (*Macoma nasuta*) [10] in Tellinidae. The presence a single 45S rDNA subterminal in a chromosome pair in *Macoma nasuta* [10] coincided in number with our results for *Bosemprella incarnata*, *Macomangulus tenuis* and *Moerella donacina* and differed with the two clusters displayed by *Serratina serrata*. In regards to location, the situation is quite different because most of the 45S rDNA clusters found in this work were subcentromeric; this was also the case for one of the three silver-stained NORs found in *Limecola balthica* [11].

Regarding 5S rDNA and histone gene clusters, no previous data was available for any of the species of Tellinidae. The presence of one or two 5S rDNA clusters has also been described in another Heterodonta family, Veneridae, in which subcentromeric, intercalary and subtelomeric locations were also found [7, 21]. The occurrence of single minor histone gene clusters, either subterminal or subcentromeric, in all tellin shells are similar to the situation found in Mactridae [28] but differs with that in Veneridae in which 1 to 4 clusters have been described [7, 21, 33].

Additionally, the simultaneous presence of both 45S and 5S rDNA clusters on the same chromosome pair in *Macomangulus tenuis* is rather scarce in bivalves; *Ruditapes decussatus*, showing overlapping 45S and 5S rDNA signals, is the only other Heterodonta species presenting this particularity [36] that was also found in three Mytilidae, *Perumytilus purpuratus* [19], *Brachidontes rodriguezii* [20] and *Mytilus trossulus* [6].

Concerning telomeric sequences, their presence exclusively at chromosome ends in *Bosemprella incarnata*, *Macomangulus tenuis*, *Moerella donacina* and *Serratina serrata* is the most common result found in bivalves [19–21, 27, 28, 32–35].

Despite their abundance and ecological importance, the systematics and taxonomy of tellin shells, mostly based on characters showing an extraordinarily high level of morphological homoplasy, is not a realistic reflection of the true phylogenetic relationships of its members and requires a profound revision [2]. This is further confirmed by the most recent molecular phylogeny of bivalves that, although including scarce tellin shell sampling, displays Tellinidae, and other Tellinoida families, as polyphiletic and highlights the need of denser taxon sampling in clades, such as Tellinoida, with poorly defined families [37]. This denser sampling has to be accompanied by an accurate determination of the specimens using, as proposed by integrative taxonomy [38, 39], a large number of characters to identify them. The information provided by the chromosomal location of the FISH signals can facilitate this task.

## Conclusion

In summary, the results presented in this work clearly demonstrated that molecular karyotype is a useful character in tellin shell identification, therefore being helpful for both taxonomic and phylogenetic purposes.

## Abbreviations

CMA: Chromomycin A3
DAPI: 4′,6-diamidino-2-phenylindole
FISH: Fluorescence in situ hybridization
NOR: Nucleolus organizing region
PCR: Polymerase chain reaction
PI: Propidium iodide
rDNA: Ribosomal DNA
rRNA: Ribosomal RNA
